# Population and Parenthood

**Published:** 1920-05-22

**Authors:** 


**THE PUBLIC WELL-BEING**—(*continued*).

### POPULATION AND PARENTHOOD.

### Some Aspects of a Difficult Problem.

The difficult and subtle race problem is tackled in many aspects in the second report of the National Birth-Rate Commission, published in a bulky volume by Messrs. Chapman and Hall on Tuesday. It contains a comprehensive consideration of the questions of population and parenthood, and coming as it does from so distinguished a body of sociologists, doctors, welfare workers and educationists, it cannot fail to attract the attention of all who are studying the subject. In fact, it is likely to be something in the nature of a standard basis for discussion over a considerable period. The Commission is still continuing its work, but it has covered already a lot of ground—some of it controversial, a good deal of it generally accepted, and all of it valuable as bringing to bear information which is the first necessity in all progress.

It laments the falling birth-rate—it is dealing with the period at the end of 1918—and strongly disagrees with Mr. Harold Cox and other witnesses that Britain could very well do with a smaller population than at present. At once it knocks up against two points upon which it is hard to pronounce a judicial opinion, so complicated are the factors, whereof many can only be forecasts. A falling birth-rate, of itself, is not necessarily a bad thing, and one has little patience with enthusiasts who get up on to the housetops and shout for more babies and more babies, as if the mere multiplication-table were all that mattered on the subject. You cannot get standardisation for the mass production of babies like Ford motor-cars, and that being so—even if Ford motor-car babies, so to say, were desirable—you must tackle the thing from a very different angle. It is more important to have five healthy babies, born and brought up in conditions fair to themselves and wise for the race, than ten weaklings, born without any chance of a healthy life, or of proper conditions of up-bringing. The report contains some cheering figures on infant mortality, and this may perhaps imply healthier babies, though it is more likely that it is due to the greater efforts which have been made in the direction of child-welfare.

It seems, at any rate, a first duty to do all that is possible for those that are brought into the world—what an enormous field that is!—before we scream for unlimited supplies. That is beginning at the wrong end. It is adding to the existing evil if we continue to pack our industrial centres with still greater numbers of people. The conditions are bad enough as it is, and will take years to improve, yet it cannot be denied that the greatest additions to the population naturally come where the population is most dense. However, it is not possible to follow that point out logically and say the fewer births the better. That is the difficulty; to advocate that would be as anti-social as advocacy of wholesale production.

### Where the Trouble Arises.

We confess we do not much care for the report's emphasis of the importance of extending the race in view of the next war. Much apprehension is expressed because of the recruiting of the race from the lower rungs of the social ladder. This is a serious factor, but it is doubtful whether it will be effectually met in the long run by any other means than raising the bottom rungs, socially and educationally. The best type of working man is a fine stock, and with increasing educational facilities there is little to fear for our future if this section continues to multiply. But it is below that level where the trouble arises, and there the task is as much that of the social reformer and the educationist as of the eugenist. Here there are to be overcome irresponsibility, squalor, ignorance, and every ill that follows from them. It is unfortunate; it is difficult; it is certainly serious. But it has to be faced squarely, and mere lamentations that the stock, which has hitherto demonstrated superior capacity—with its superior chances—has ceased to contribute its fair share, carry us nowhere. All means, certainly, should be used to impress upon this section of the community its responsibilities—as a matter of fact, it is largely its consciousness of them that keeps down the numbers—but at the same time a strenuous and sustained effort must be made to make those who are producing in the largest numbers worthy of so

**THE PUBLIC WELL-BEING**—(*continued*).

serious a privilege. On the other hand, it cannot be too strongly asserted that those who refuse to bring children into the world for reasons of luxury and selfishness deserve nothing but contempt.

The report shows considerable difference of opinion on the subject of voluntary restriction of the size of families. As the Commission has a membership of forty-one, its attitude may be indicated as follows:—For certain forms of artificial restriction, 4; against any form of artificial restriction 15; expressing no final opinion, 22.

Point of General Agreement.

A point upon which all the Commissioners found themselves in agreement was that, in view of the spread of information upon this subject, it is essential that instruction should be given, especially to young people, in the laws of sex hygiene, in the wrongfulness of sex immorality, in the prevalence and danger of venereal disease, in the right and healthy use of the state of marriage, in the immorality of abortion, in the duties and privileges of parenthood, and in the value of family life to the nation and human race.

Other subjects dealt with are alcoholism, illegitimacy, and the milk supply. The problem of the illegitimate child is regarded as "a national problem urgently calling for solution," the facts being that:

The number of illegitimates shows no sign of decreasing.

The mortality amongst illegitimate is double that of legitimate-born children.

The nation cannot acquiesce in the destructio[illegible] of children, legitimate or illegitimate.

The Commission's view is that there is urgent need for more accommodation for the unmarried mother and her child, and for more efficient guardianship of the child. Carefully supervised hostels should be set up in which mothers can care for their children, and at the same time learn a trade or become efficient in domestic work, while assured that their little ones are safe. These hostels should be homes to which the mother could return at night. Such are the lines followed by the most successful of existing schemes, though they are nothing near an approach to adequacy in point of numbers. On the question of venereal disease the report commends disinfection, and expresses the opinion that the time has arrived for the State to make a trial of compulsory notification and treatment, provided there is no return to the principle or practice of the Contagious Diseases Act.

